# *In vitro* knockdown of *Ts*DNase II-7 suppresses *Trichinella spiralis* invasion into the host’s intestinal epithelial cells

**DOI:** 10.1371/journal.pntd.0011323

**Published:** 2023-06-08

**Authors:** Jing Wang, Xuemin Jin, Chengyao Li, Xinhui Chen, Yanfeng Li, Mingyuan Liu, Xiaolei Liu, Jing Ding

**Affiliations:** State Key Laboratory for Zoonotic Diseases, Key Laboratory for Zoonosis Research of the Ministry of Education, Institute of Zoonosis, and College of Veterinary Medicine, Jilin University, Changchun, China; Universidad del Valle de Guatemala, GUATEMALA

## Abstract

*Trichinella spiralis* (*T*. *spiralis*) adult-specific deoxyribonuclease II-7 (*Ts*DNase II-7), a member of the DNase II-like nuclease family with no DNase II activity, was identified in the excretory–secretory (ES) products of adult worms (AWs). However, its biological functions are still unclear. Our previous study revealed that *Ts*DNase II-7 is located around the infection site in the intestinal tissue, speculating that it was involved in the *T*. *spiralis* invasion of host intestinal epithelial cells (IECs). This study aimed to use RNA interference to verify our speculation that *Ts*DNase II-7 in 3-day old adult *T*. *spiralis* (Ad3) plays a role in intestinal invasion. *Ts*DNase II-7-specific small interfering RNAs (siRNAs) were delivered into muscle larvae (MLs) to knockdown *Ts*DNase II-7 expression by electroporation. Twenty-four hours later, the MLs transfected with 2 μM siRNA-841 exhibited decreased in *Ts*DNase II-7 transcription and expression as compared to the control MLs. The knockdown of *Ts*DNase II-7 expression did not affect ML viability, and the low expression of *Ts*DNase II-7 still maintained in Ad3 recovered from *Ts*DNase II-7-RNAi-ML infected mice, resulting in a weakened ability of Ad3 to invade intestinal epithelial cells (IECs). These results indicated that knockdown of *Ts*DNase II-7 gene expression via RNA interference (RNAi) suppressed adult worm invasion and confirmed that *Ts*DNase II-7 plays a crucial role during the intestinal phase of *T*. *spiralis* infections, which provided new candidate for vaccine development of *T*. *spiralis*.

## Author summary

*T*. *spiralis* adult-specific DNase II-7, a member of the DNase II-like nuclease family with no DNase II activity, whose biological functions are still unknown. We previously found that *Ts*DNase II-7 is located around the infection site in the intestinal tissue, speculating that it was involved in the *T*. *spiralis* invasion of host intestinal epithelial cells. In this study, the results showed that *Ts*DNase II-7 is highly transcribed and expressed in the adult stage of *T*. *spiralis*, and promoted *T*. *spiralis* penetration into intestinal epithelial cells, while knockdown of *Ts*DNase II-7 gene expression by siRNA-*Ts*DNase II-7 impaired worm invasive ability. Our study demonstrated the essential role of *Ts*DNase II-7 in invasion and development, highlighting its potential as a molecular target for vaccine development against *T*. *spiralis* infection.

## Introduction

Trichinellosis is mainly caused by infection of *Trichinella spiralis* (*T*. *spiralis*), which is a major foodborne parasitic nematode prevalent in more than 150 mammalian species worldwide [1]. Humans acquire trichinellosis by ingesting raw or semiraw meat and meat products from swine, horse, dogs and game animals contaminated by the muscle larvae (MLs) of *T*. *spiralis* [2–3]. Trichinellosis affects human health and increases food safety risks, causing substantial economic, social, and public health impacts in endemic countries. After *T*. *spiralis* infection and digestion by enzymes in the stomach, the infective MLs migrate to the intestine and develop into intestinal infective larvae (IILs) [4]. IILs grow into sexually mature adult worms (AWs) after undergoing four molts. The AWs live in the intramulticellular niche of the columnar epithelium of the intestinal mucosa. Subsequently, gravid female AWs produce newborn larvae (NBLs), which are distributed throughout the body via lymphatic/blood circulation. Next, the NBLs migrate and incapsulate the skeletal myocytes of hosts to complete their life cycle [5]. The AW stage is a critical stage of the *T*. *spiralis* lifecycle, and the intestinal epithelium is the primary barrier against *T*. *spiralis* infection and the crucial site for the interaction between the intestinal nematode and host [6–7]. If the colonization process of adult worms in intestinal epithelial cells is affected, the occurrence and progression of trichinellosis will be prevented. Therefore, screening proteins that play a crucial role in intestinal invasion is critical in the preventing and controlling of trichinellosis.

*T*. *spiralis* excretory/secretory (ES) products, which are a complex mixture of constituents with various biological functions, such as larval invasion, molting, digestion and proteolysis, are essential for the long-term survival of *T*. *spiralis* by helping it overcome the host defense system and interact with the surrounding host intestinal epithelium during infection [8–9]. Wang et al. found that anti-ES serum inhibited *T*. *spiralis* invasion into the gut epithelium and larval development, indicating that *T*. *spiralis* ES proteins may include invasion-associated components, such as aspartic protease, glutathione S-transferase and novel gut-specific cysteine proteases [10–13]. In view of the importance of the invasion process of *T*. *spiralis*, more proteins that play a role in this process need to be identified.

Compared with Deoxyribonuclease II (DNase II) from other species, approximately 125 members of the DNase II-like protein family of *T*. *spiralis* have been documented [14]. In fact, most DNase II family members have striking sequence conservation in the 5-mer DHSKW surrounding the active histidine residue [15]. However, the active sites of *T*. *spiralis* DNase II family members are altered, with histidine replaced entirely by lysine or serine according to sequence analysis. Among *T*. *spiralis* DNase II-like protein family members, *Ts*DNase II-7 has been proved to have no enzyme activity, so it is impossible for this protein to play a biological function through nuclease activity. In order to explore the actual biological function of *Ts*DNase II-7, we performed localization analysis of the protein and found that it is located abundantly in the teguments of AWs and secreted into the small intestines. Since the key to complete lifecycle of *T*. *spiralis* in the host and the successful parasitism is to invade the intestinal epithelium, we postulated that in the early stages of *T*. *spiralis* infection, damage to the intestinal epithelium was also in direct contact with DNase II-like protein family members secreted into the intestine. Therefore, the purpose of this study was to ascertain the functions of *Ts*DNase II-7 during the process of invasion of the host intestine, so as to provide new candidates for the vaccine development of *T*. *spiralis*.

## Materials and methods

### Ethics statement

The animals were treated in strict accordance with the National Institutes of Health guidelines (publication no. 85–23, revised 1996). The animal protocols were approved by the Ethical Committee of Jilin University, affiliated with the Provincial Animal Health Committee, Jilin Province, China (Ethical Clearance number IZ-2009-08).

### Worms, experimental animals, and cells

The *T*. *spiralis* isolate ISS 534 was maintained by serial passage in ICR mice at the Institute of Zoonoses of Jilin University. ICR mice (females; aged 6–8 weeks; 15–20 g) and female Wistar rats were obtained from Norman Bethune University of Medical Science (NBUMS), China. Rat small intestinal epithelial IEC-6 cells (purchased from American Type Culture Collection, ATCC; Manassas, VA, USA) were grown in Dulbecco’s minimum essential medium (Gibco) with 10% FBS, and they were susceptible to invasion by *T*. *spiralis*. Chinese hamster ovary (CHO) cells (purchased from ATCC; Manassas, VA, USA) were grown in Dulbecco’s minimum essential medium (Gibco) with 10% FBS. All cells were grown in a humidified 5% CO_2_ atmosphere at 37°C.

### Sequence analysis

In the current study, the complete *Ts*DNase II-7 cDNA sequences were obtained from the GenBank database (GenBank: AAY32322.1) and analyzed using NCBI utilities and BLAST searches (http://www.ncbi.nlm.nih.gov/BLAST) [16] for DNase II homologs. The molecular weight and theoretical pI of *Ts*DNase II-7 were predicted using the online website ExPASy (https://web.expasy.org/protparam) [17]. The signal peptide, transmembrane domain, and N-linked glycosylation sites were analyzed using the SignalP program (https://services.healthtech.dtu.dk/services/SignalP-5.0) [18], TMHMM program (https://services.healthtech.dtu.dk/services/TMHMM-2.0) [19] and NetNGlyc program (https://services.healthtech.dtu.dk/services/NetNGlyc-1.0) [20], respectively. The conserved domains were predicted using the Conserved Domain Database of National Center for Biotechnology Information (NCBI) (http://www.ncbi.nlm.nih.gov/Structure/cdd/wrpsb.cgi). The amino acid sequences were aligned with DNase II-like protein family genes of other *Trichinella* species and other organisms using Clustal W and DNAMAN version 6.0 (Lynnon Biosoft, USA). Phylogenetic trees based on the *T*. *spiralis* DNase II homologous sequences were constructed with DNAMAN version 6.0 software (Lynnon Biosoft, USA).

### Cloning of DNase II cDNA, plasmid construction and sequence analysis of recombinant expression plasmids

Bovine DNase II cDNA was obtained by PCR using of bovine spleen cDNA with a pair of primers carrying *Bam*HI and *Eco*RI restriction enzyme sites (5’-GGATCCATGGCCACGCTGAGCTCGCTGC-3’ and 5’-GAATTCTCAATGATGATGATGATGATGAGTCCACAGTCCTGC-3’). Forward primers (5’-GGATCCGTTGACTTCCAATGTTTCCAAGATG-3’, *Bam*HI site underlined) and reverse primers (5’-GAATTCTTAATGATGATGATGATGATGGGTGATGCATCCAA-3’, *Eco*RI site underlined) were used to amplify the *Ts*DNase II-7 gene cDNA attached with a His tag at the 3’-end. Target cDNA fragment of *Ts*DNase II-7 was ligated into pET-28a (+) or pcDNA 3.4 to construct the recombinant plasmid pET28a/*Ts*DNase II-7 or pcDNA 3.4-*Ts*DNase II-7. In the same way, target cDNA fragment of bovine DNase II was ligated into pcDNA 3.4 to construct the recombinant plasmid pcDNA 3.4-bovine DNase II. Clones containing inserts of the expected size were selected by restriction digestion and gene sequence were identified by DNA sequence analysis (Sangon Biological Co., Ltd., Shanghai, China).

### Expression and purification of recombinant *Ts*DNase II-7 (r*Ts*DNase II-7) and rabbit antiserum

The correctly constructed expression plasmids pET28a/*Ts*DNase II-7 were transformed into *Escherichia coli* BL21 (DE3) (Novagen, USA) for expression. The positive clone verified by DNA sequencing was inoculated in 10 mL of LB medium with Kanamycin (100 μg/mL) and incubated at 225 rpm at 37 °C overnight. Then 1 L of LB medium supplemented with the same antibiotics were mixed with 10 mL of the overnight culture. Temperature at 37 °C with 225 rpm until an OD of 0.5–0.6 was achieved at 600 nm. After induction of the expression with 1 mM isopro-pyl-β-D-thioga-lactopyranoside (IPTG) at 37°C for 6 h, bacterial precipitate was harvested after 30 min ultrasonic crushing on ice using centrifugation at 3,000 g for 25 min and subjected to protein purification. The r*Ts*DNase II-7 was purified by Ni-affinity chromatography (Qiagen, California) according to the manufacturer’s instructions.

A rabbit was subcutaneously immunized with approximately 500 μg of purified r*Ts*DNase II-7 (1 mg/mL) emulsified with Freund’s complete adjuvant (FCA; Sigma, USA) and boosted four times at a two-week interval. Fourteen days after the final immunization, blood samples were taken from immunized rabbit heart and a polyclonal antibody against r*Ts*DNase II-7 at a titer of 1:256,000 was analyzed by ELISA. Affinity-purified antibodies were used for subsequent Western blot and immunofluorescence assays (IFAs).

### Expression of *Ts*DNase II-7 in CHO and DNase activity assays

Before transfection, CHO cells were cultured in ExpiCHO Expression medium (Thermo Fisher, USA) at a density of 4–6 million cells/mL for more than two passages. On the day of transfection, the cells were at a density of 7–10 million cells/mL (95–99% viability). Transfections were performed using the ExpiCHO Expression System Kit (Thermo Fisher, USA) according to the manufacturer’s protocol. Bovine plasmid pcDNA 3.4-DNase II transfection was performed as a negative control experiment. Briefly, ExpiFectamine CHO transfection reagent and plasmid pcDNA 3.4-DNase II were separately diluted in OptiPRO SFM. The ExpiFectamine CHO-DNA-OptiPRO complex was then added to the cells. One day after continuous shaking cell culture (37°C, 5% CO_2_, 125 rpm), the transfected cells were supplemented with 150 μL of ExpiFectamine CHO enhancer and 4 ml of ExpiCHO feed. The cell culture supernatant was collected 12 days after transfection (60–75% viability) and filtered through a 0.22 μm filter. The protein samples were affinity-purified on an ÄKTA UPC 900 FPLC device using a HisTrap HP column (GE Healthcare, Piscataway, NJ) with buffer containing imidazole (50 mM, 100 mM, 500 mM) and then were detected by Western blot. Anti-*Ts*DNase II-7 was used as the primary antibody.

DNase II activity was analyzed by agarose gel electrophoresis and the diffusion method. Briefly, 1 μg of substrate DNA (salmon sperm DNA; Sigma Chem., USA) was incubated at 37°C for 1 h in total reaction volume of 20 μL contained 10 μL of 2× buffer (pH 5), [2 μg of BSA, 2 μg of r*Ts*DNase II-7 or 100 units of commercialized bovine DNase II], and DEPC-treated water up to a final volume. After stopping the reaction, the samples were subjected to electrophoresis on a 1% agarose gel. Radial diffusion in gel method: 1% melted agarose (55°C-60°C) containing 0.5 μg/μL of substrate DNA was poured on gel diffusion plates. Once the gel had solidified, wells, each 4 mm in diameter, were cut out of the gel, and filled with [100 units of commercialized bovine DNase II, 2 μg of ExpiCHO-expressed DNase II protein sample (*Ts*DNase II-7 or bovine DNase II) or 2 μg of BSA], and the plates were incubated at 37°C for 1 h.

### SiRNA preparation

Chemically synthesized RNA interference molecules for *Ts*DNase II-7 were designed according to the sequencing results (comparison between sample cDNA and GenBank: AY963701.1) and purchased from Sangon Biotech (Shanghai, China). Three *Ts*DNase II-7 specific siRNAs, siRNA-841 (5’-GCACAUUAAAAGUGGAAAATT-3’), siRNA-382 (5’-CAGCAAAGUALU UCCCAAATT-3’) and siRNA-1030 (5’-GCAAAGAAGCAGCGGCCUUTT-3’) were used in the present study. A FAM-labeled control siRNA (Sangon Biotech, China) carrying a scrambled sequence (5’-UUCUCCGAACGUGUCACGUTT-3’) was applied as a specificity control and used to evaluate transfection efficiency.

### Collection of worms at distinct stages and protein preparation

The mice experimentally infected with 250 MLs of the *T*. *spiralis* strain ISS 534 were killed at 35 days postinfection (dpi) and digested at 37°C for 2 h with artificial digestion (1% pepsin/HCl). The MLs were counted by stereomicroscopy, and AWs were recovered from the intestines of mice infected with 1,200 MLs at 1 dpi (Ad1), 2 dpi (Ad2), 3 dpi (Ad3), 4 dpi (Ad4), and 5 dpi (Ad5). NBLs were recovered from Ad5 incubated in RPMI-1640 medium (Gibco, US) at 37°C in 5% CO_2_ for 24 h, purified by filtration through a 100 μm filter (Millipore, US) and centrifuged at 2,000 r for 5 min. Crude extracts of worms of ML and Ad3 were prepared. Briefly, the worms were lysed in ice-cold RIPA buffer (Beyotime, China) with a protease inhibitor cocktail (Beyotime, China), incubated on ice for 30 min and centrifuged at 12,000 rpm for 20 min to collect the supernatants.

### Delivery of siRNA into the ML

The MLs were collected from the mice of each group by artificial digestion as described above and were washed three times with sterile PBS. For electroporation, 2,500 fresh larvae were suspended in 250 μL of electroporation buffer containing 2 μM of specific siRNA or control siRNA and 2 μL of Lipofectamine 2000 (Gibco, US). The worms were transfected via electroporation using a Bio-Rad Gene Pulser Xcell Electroporation System (800 V, 200 Ω, 25 μF), and RPMI 1640 culture medium (100 units/mL penicillin and 100 mg/mL streptomycin) was added up to 500 μL and cultured at 37°C and 5% CO_2_ for 24 h. Ad3 was obtained at 3 dpi from the intestines of mice infected with siRNA 841-treated ML.

### Quantitative real-time PCR

To observe the transcription of the *Ts*DNase II-7 gene at different *T*. *spiralis* developmental stages, total RNA was extracted from the AW (Ad1, Ad2, Ad3, Ad4, Ad5), NBL and ML. To detect whether siRNA can affect *Ts*DNase II-7 transcription, total RNA from siRNA-treated and control ML or Ad3 was extracted with RNAiso Plus (TaKaRa, Japan). Subsequently, of each RNA sample, 1 μg was transcribed into first-strand cDNA using the All-in-One First-Strand cDNA Synthesis SuperMix (TransGen, China). The *Ts*DNase II-7 transcription level was determined by qPCR using TransStart Green qPCR SuperMix (TransGen, China) and Applied Biosystems StepOnePlus Real-Time PCR System (ThermoFisher Scientific, USA). The specific primers for qPCR to amplify the *Ts*DNase II-7 gene were 5’-CCGCCCAGCCATTCTACGATTC-3’ and 5’-AAGTCCTTATGCCATCACCAGAAGC-3’. The GAPDH gene (GenBank no. AF452239.1) served as the control of the housekeeping gene. The primers were designed as follows: forward, 5’-GTGCTGATTACGCTGTTG-3’; reverse, 5’-CTAAGCCATTGGTAGTGC-3’. Reactions were performed with 40 cycles of 3 s at 95°C, and 30 s at 60°C. Data were analyzed using the comparative Ct (2^−ΔΔCt^) method. Three independent assays were conducted, and each sample was analyzed in triplicate.

### Western blot analysis

The protein samples of siRNA-treated and control ML or Ad3 were prepared as described above. The total protein concentrations were determined using the BCA assay (Beyotime, China), and equal amounts of protein (35 μg) from each treated worm group were separated by 12% sodium dodecyl sulfate–polyacrylamide gel electrophoresis (SDS–PAGE) and then transferred onto a polyvinylidene difluoride (PVDF) membrane (Roche, Germany). The membranes were blocked with 5% skim milk in TBST with 0.05% Tween-20 and then incubated with rabbit anti-r*Ts*DNase II-7 antibody (1:400) or anti-GAPDH antibody (ab8245; Abcam, UK) overnight. After that, the membrane was washed five times with TBST and incubated with an anti-rabbit IgG, HRP-linked antibody (#7074; Cell Signaling Technology, USA) in blocking buffer for 2 h at room temperature. Signals were detected using the Pierce ECL Western Blot Substrate (ThermoFisher Scientific, USA) and UVP Chemstudio (Analytik Jena, Germany) according to the manufacturers’ instructions.

### IFA of *Ts*DNase II-7 binding with IEC and cellular localization

To observe the effect of mechanism-RNA interference (RNAi) on the ability of Ad3 to invade IECs, IECs were grown to approximately 90% confluence on glass coverslips in DMEM in 24-well plates overnight. Each cell monolayer was overlaid with approximately 100 Ad3 worms treated with siRNA-841 or control siRNA suspended in 80 μL of semisolid medium (DMEM/10% FBS containing 3.75% agarose). The 24-well plate was incubated at 37°C in 5% CO_2_ for 2 h. The monolayer cell nuclei were dyed with propidium iodide (PI) (0.03 mg/mL in DMEM-50% FBS) and incubated at 4°C for 30 min. This protocol for PI staining can be used to discriminate dead cells, in which plasma membranes become permeable regardless of the mechanism of death. After being washed twice with DMEM-10% FBS, the monolayer was fixed with 4% paraformaldehyde and then incubated for 1 h at 37°C with anti-r*Ts*DNase II-7 immune serum (1:400). Subsequently, they were reacted for 40 min at 37°C with the secondary antibody conjugated to Alexa Fluor 488 goat anti-rabbit IgG H+L (1:1,000; Abcam, USA), followed by visualization using a fluorescence microscope (Olympus, Japan). Additionally, Ad3 invasion of IECs was observed using an inverted microscope (Olympus, Japan).

### Localization of *Ts*DNase II-7 by IFA in intestinal tissue from *T*. *spiralis*-infected mice

Small intestines collected from *T*. *spiralis*-infected mice were embedded in paraffin and then sliced into 5 μm thick sections after fixation with 4% paraformaldehyde. The tissue sections were treated with 5% BSA to block any nonspecific binding and then were incubated with a 1:400 dilution of rabbit-anti-r*Ts*DNase II-7 antiserum (experimental group) and *T*. *spiralis*-uninfected rat serum (negative control group) as the primary antibody at 4°C. After 12 h of incubation, the slides of both groups were incubated with the secondary antibody conjugated to Alexa Fluor 488 goat anti-rabbit or anti-mouse IgG H+L (1:1,000; Abcam, USA) at room temperature for 1 h. Hoechst 33342 (Sigma, USA) was used to stain the nuclei, and anti-fade fluoromount medium (Beyotime, China) was used before visualization under a laser scanning confocal microscope (Olympus, Japan). Images were obtained with a flat section and Z-stack using the Olympus FV10-ASW software v02.01.03.10 (Olympus Corporation).

### Infectivity of siRNA-treated worms to IECs *in vitro*

Thirty ICR mice were divided into three groups (10 mice each). Each group was orally inoculated with 1,200 *T*. *spiralis* ML electroporated with siRNA-841, control siRNA, or PBS. Ten mice from each group were sacrificed at 3 dpi, and the adult worms were collected from the intestine of infected mice and counted. To identify whether *Ts*DNase II-7 participated in *T*. *spiralis* invasion of IECs, Ad3 treated with siRNA-841 or control siRNA was used in the invasion test. Semisolid DMEM mixed with 100 Ad3 worms was added to the IEC monolayer. After incubation at 37°C for 2 h, Ad3 invasion of IECs was observed using an inverted microscope (Olympus, Japan). The Ad3 worms invading and migrating in the IEC monolayer were counted as invaded worms, whereas the worms that were still suspended in the medium and located on the IEC surface were counted as noninvaded worms. The invasion rate of siRNA-treated Ad3 was compared with those of the control siRNA-treated group and untreated group.

### Statistical analysis

Data were analyzed using GraphPad Prism 8 software for Windows. The differences between the treatment and control groups were analyzed using one-way analysis of variance (ANOVA). Data were expressed as the mean ± SD (standard deviation) of at least three repeated experiments. *P* values < 0.05 were considered statistically significant. In all cases, p-values were expressed as * *P* < 0.05, ** *P* < 0.01 and *** *P* < 0.001.

## Results

### Phylogenetic analysis and bioinformatic analysis of *Ts*DNase II-7 sequences

**Fig 1:**
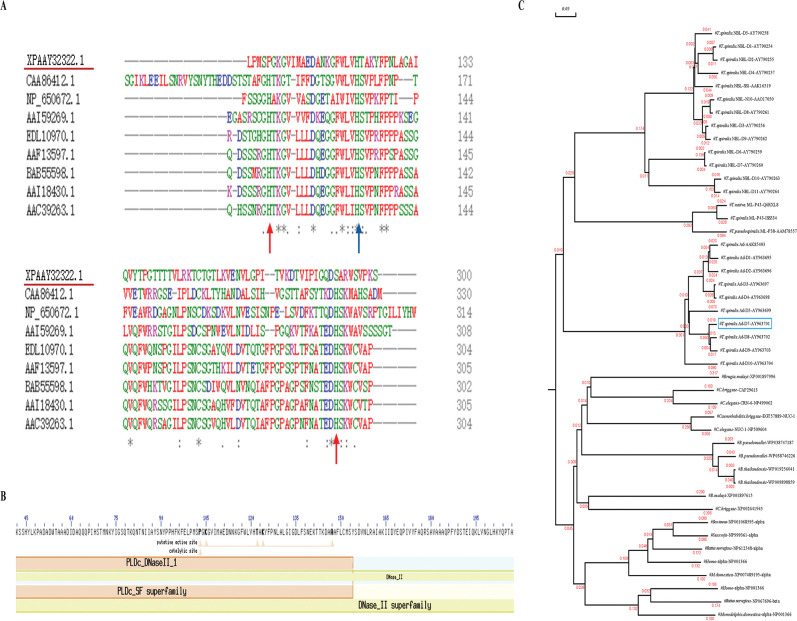
Phylogenetic analysis and amino acid sequence alignment of DNase II from *T*. *spiralis* (AY963701) and other organisms. (A) Multiple sequence alignment of *Ts*DNase II-7 and homologs from other organisms. The putative active sites are indicated by arrows. (B) Motif searching of amino acids completes the *Ts*DNase II-7 sequence. (C) Phylogenetic tree of *T*. *spiralis* DNase II and its homologs in other species. The phylogenetic tree was constructed by DNAMAN, and sequences and their accession numbers are shown on the graph. The blue box indicates adult-specific *Ts*DNase II-7.

Based on the results obtained from the prediction software, we revealed that the complete cDNA sequence of the *Ts*DNase II-7 gene was 1,161 bp, encoding 348 amino acids with the characteristics of *Ts*DNase II-7, including a DNase II superfamily domain and no transmembrane helix or N-linked glycosylation sites (Fig 1B). The predicted MW and pI of *Ts*DNase II-7 were 36.09 kDa (without signal peptide) and 6.57, respectively. The signal peptides were located at 1–18 aa. Clustal W analysis of the sequence revealed that the putative active sites are shown in comparison to those of other species (Fig 1A). Significant conservation of the active histidine residue has changed in *T*. *spiralis*, indicating that substitutions of histidine residues may allow changes in canonical DNase II activity. A total of 45 DNase II sequences from various organisms, including the *Trichinella* group (*T*. *spiralis*, *T*. *pseudospiralis* and *T*. *nativa*), *Brugia malayi*, *Caenorhabditis briggsae*, *Caenorhabditis elegans*, *Burkholderia pseudomallei*, *Burkholderia thailandensis*, *Bos taurus*, *Sus scrofa*, *Rattus norvegicus*, *Monodelphis domestiaca* and *Homo* were used to reconstruct the phylogenetic tree. Phylogenetic analysis of *T*. *spiralis* Ad-D7-AY963701 using the DNAMAN program showed that DNase II of the *Trichinella* group is on the same separate branch (Fig 1C).

### DNase II protein samples were correctly expressed in eukaryotic cells

**Fig 2:**
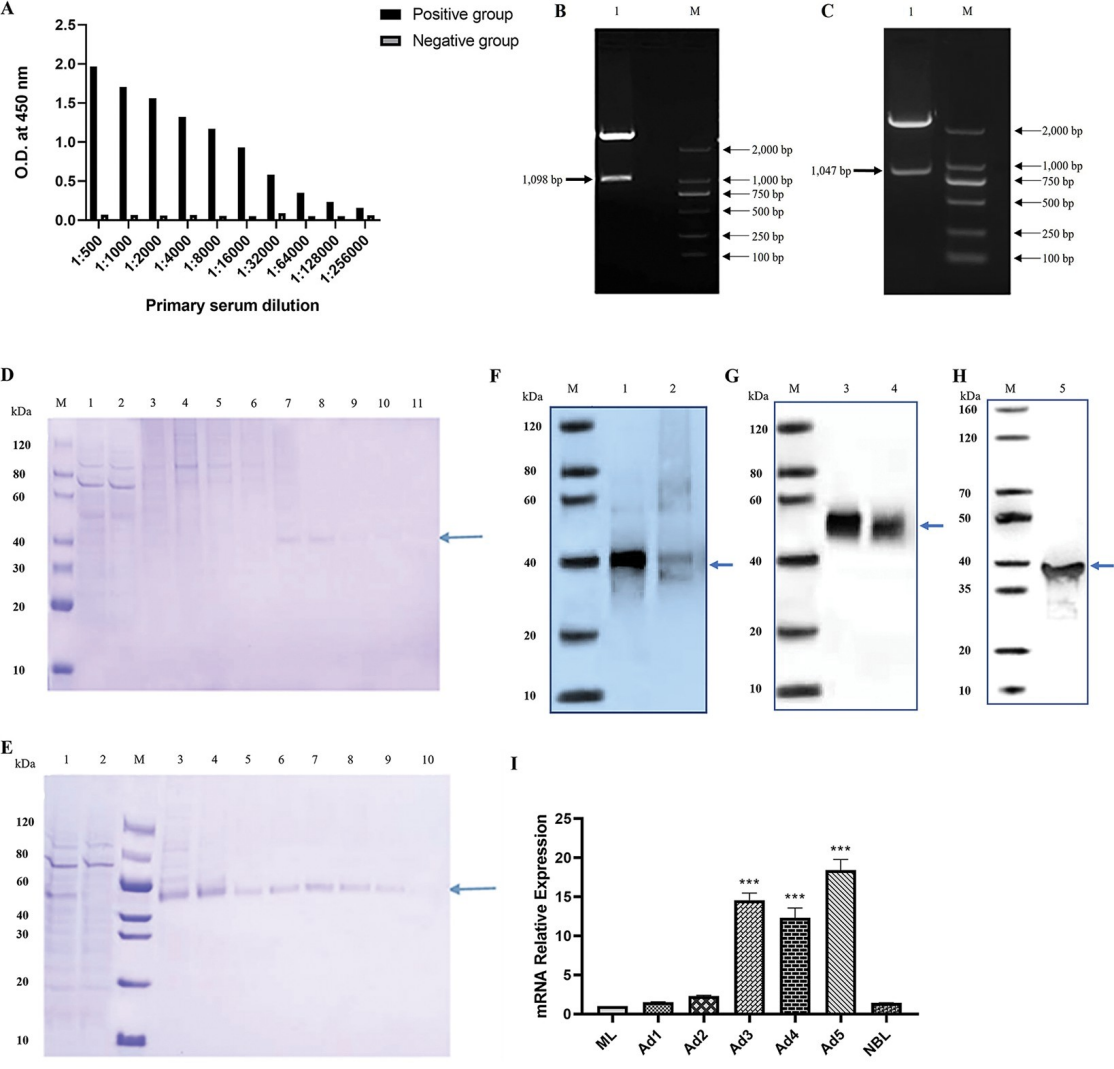
Anti-DNase II antibody detects recombinant DNase II protein expression in the mammalian ExpiCHO cell system. (A) Analysis of antibodies from rabbit immunized with r*Ts*DNase II-7 by ELISA. (B-C) Agarose gel electrophoresis of double enzyme digestion identification of pcDNA 3.4-DNase II recombinants. (B. Bovine DNase II M: DL2000 DNA Marker, Lane 1: pcDNA 3.4-DNase II (*Bam*HI and *Eco*RI); C. M: DL2000 DNA Marker, Lane 1: pcDNA 3.4-DNase II (*Bam*HI and *Eco*RI). The specific band is indicated by the arrow. (D-E) The *Ts*DNase II-7 protein (D) and bovine DNase II (E) were eluted with different concentrations of imidazole solutions (50 mM, 100 mM, 500 mM). (1: supernatant after centrifugation; 2: effluent after supernatant incubation with His-Trap 3–4: elution of 50 mM imidazole solution; 5–6: elution of 100 mM imidazole solution; 7–10: elution of 500 mM imidazole solution). (F-H) DNase II protein expression in CHO cells was detected by anti-r*Ts*DNase II-7 polyclonal antibody after 12 days of culturing using Western blot. Bovine DNase II and *Ts*DNase II-7 were analyzed using anti-HIS antibody (Lanes1, 2 denatured and nondenatured *Ts*DNase II-7; Lanes 3, 4 denatured and nondenatured bovine DNase II) or anti-r*Ts*DNase II-7 antibody (Lane 5: denatured *Ts*DNase II-7). Arrowheads indicate bands recognized by only anti-His or anti-r*Ts*DNase II-7 antibody. (I) qPCR analysis of *Ts*DNase II-7 mRNA levels at different *T*. *spiralis* stages. *Ts*DNase II-7 mRNA from ML, 1-day, 2-day, 3-day, 4-day, 5-day AW and NBL was isolated and amplified by qPCR. The *Ts*DNase II-7 transcription level was calculated according to the Ct (2^-ΔΔCt^) method. The fold change of *Ts*DNase II-7 genes normalized to GAPDH served as a normalizer gene. Three repeats for each sample were performed. *** *P* < 0.001 compared with the ML stage.

Serum specific anti*-*r*Ts*DNase II-7 titers at 14 days after the final immunization of rabbit with 1 mg/mL r*Ts*DNase II-7 each time were measured by ELISA. As shown in Fig 2A, the titer of the polyclonal antibody against r*Ts*DNase II-7 was 1:256,000, suggesting that r*Ts*DNase II-7 has high immunogenicity.

Total RNA was extracted from bovine spleen and *T*. *spiralis* Ad3, and different DNase II amplified fragments were assayed using RT–PCR. The presence of different DNase II genes (1,098 bp in bovine DNase II; 1,047 bp in *Ts*DNase II-7) was confirmed using double digestion (*Bam*HI and *Eco*RI) of the pcDNA 3.4-DNase II recombinant vector (Fig 2B and 2C). The pcDNA 3.4 vector has an effective promoter applied to obtain high levels of recombinant protein expression in mammalian cells. Next, the accumulation of DNase II protein in the ExpiCHO Expression culture medium was assessed. After purification using a HisTrap HP column and SDS–PAGE gel extraction, purified samples were identified by SDS–PAGE and Western blot. The molecular mass of the DNase II proteins was approximately 40 kDa (Fig 2D and 2E), and the recombinant protein was recognized by the anti-His antibody or anti*-*r*Ts*DNase II-7 antibody (Fig 2F–2H).

### *Ts*DNase II-7 mRNA levels were higher in adult stage of *T*. *spiralis*

The qPCR findings showed that *Ts*DNase II-7 mRNA was expressed at all developmental stages (ML, AW, and NBL) (Fig 2I). The *Ts*DNase II-7 mRNA levels in the Ad3, Ad4 and Ad5 stages were higher than those in the ML stage (*P* < 0.01), whereas the levels in the Ad1, Ad2, and NBL stages were no difference compared with those in the ML stage (*P* > 0.05). Additionally, the *Ts*DNase II-7 mRNA level in Ad3 was significantly higher than those in all AW stages except Ad5 (*P* < 0.01).

### *Ts*DNase II-7 lacked DNase II activity

**Fig 3:**
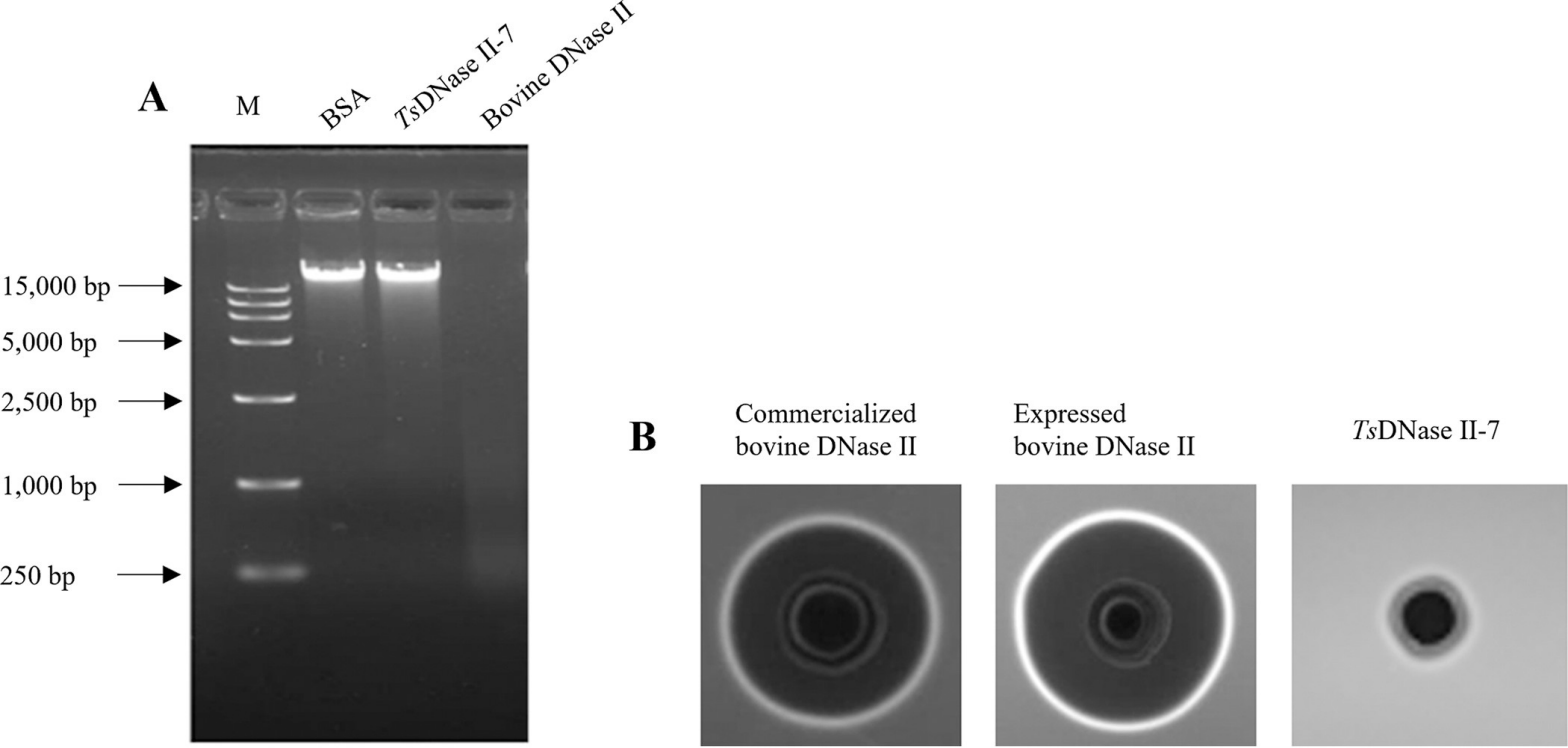
Cleavage of substrate DNA by two different DNase II enzymes. (A) The activities of bovine DNase II and *Ts*DNase II-7 after coincubation of purified protein with substrate DNA were assayed using agarose gel electrophoresis (37°C, pH = 5, 1 h). (B) Equal amounts of different DNase II enzymes (2 μg of ExpiCHO-expressed DNase II protein samples, 100 units of commercialized bovine DNase II) and 2 μg of BSA were loaded on a 1% agarose gel (containing substrate DNA) and analyzed for DNase II activity under conditions known to activate DNase II activity, as described under the agar diffusion method (37°C, pH = 5, 1 h). 2 μg of BSA (negative control); 100 units of commercialized bovine DNase II and 2 μg of ExpiCHO-expressed bovine DNase II (positive control).

Although this protein is predicted to have no DNase II activity based on sequence homology, we need to verify whether *T*. *spiralis* DNase II has nuclease activity through co-incubation of *Ts*DNase II-7 (purified by the above method) with salmon sperm DNA. The *Ts*DNase II-7 protein could not catalyze the degradation of salmon sperm DNA (final DNA concentration of 0.5 μg/μL) and appeared as a narrow band similar to bovine serum albumin (2 μg BSA, negative control) under UV light (Fig 3A). As shown in the substrate gel assay (Fig 3B), *Ts*DNase II-7 protein had no DNase II activity compared with bovine DNase II (positive control). Additionally, although these results were merely semi quantitative, they strongly support that the *T*. *spiralis* adult-specific DNase II-7 protein has no DNase II activity using two different methods (according to methods mentioned above).

### Immunolocalization of *Ts*DNase II-7 on the teguments of whole *T*. *spiralis*

**Fig 4:**
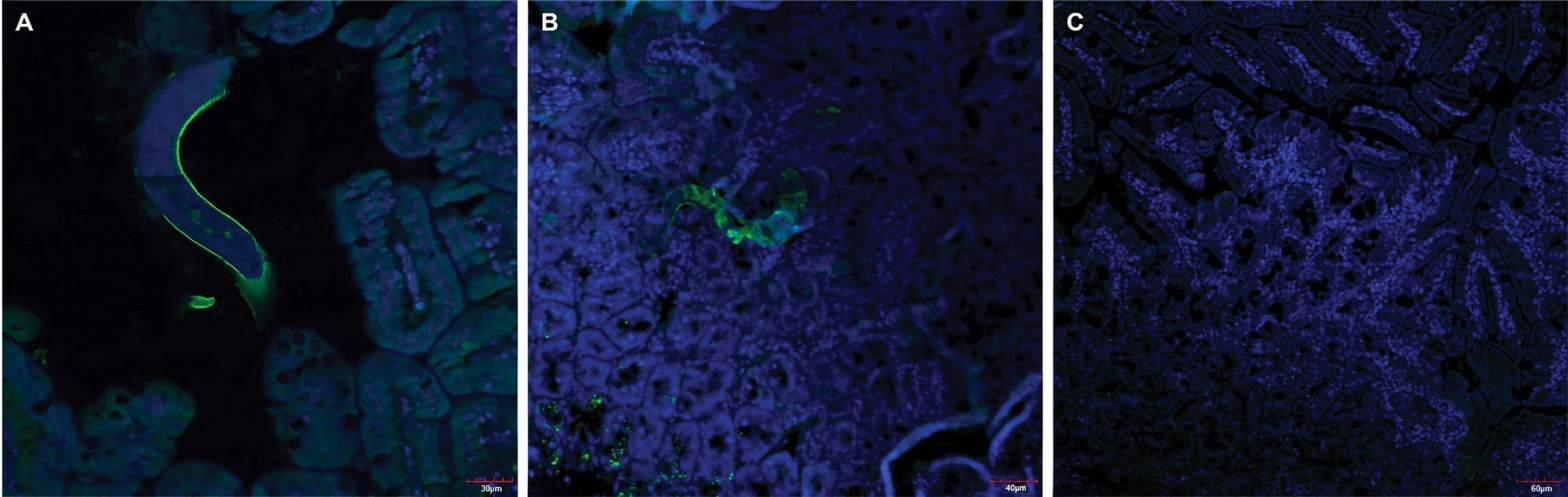
Immunolocalization of *Ts*DNase II-7 at surface of whole *T*. *spiralis* at 3 dpi. (A-C) Sections of substrate DNA from the intestines of infected mice reacted with anti-r*Ts*DNase II-7 serum by IFA. Immunostaning was observed at the cuticle and internal organs of AWs at 3 dpi. AW at 3 dpi reacted with the serum of r*Ts*DNase II-7-immunized rabbit (A-B) as a positive group; AW at 3 dpi did not recognize by uninfected mouse serum (C) as a negative control. A, scale bars = 30 μm, zoom×1.8; B, scale bars = 40 μm, zoom×1.4; C, scale bars = 60 μm, zoom×1.0.

IFA was performed using small intestinal sections from *T*. *spiralis*-infected mice at 3 dpi to localize *Ts*DNase II-7. Nuclei were stained with Hoechst 33342 (blue), and the target protein was labeled with secondary antibodies conjugated to Alexa Fluor 488 (green). *Ts*DNase II-7 exhibited prominence on the surface of Ad3 worm bodies as shown in Fig 4A and 4B, and it was also found to be secreted into the intestine. No protein was observed in the negative control group (Fig 4C).

### *Ts*DNase II-7 mRNA and protein expression levels were reduced after knockdown of *Ts*DNase II-7 gene

**Fig 5:**
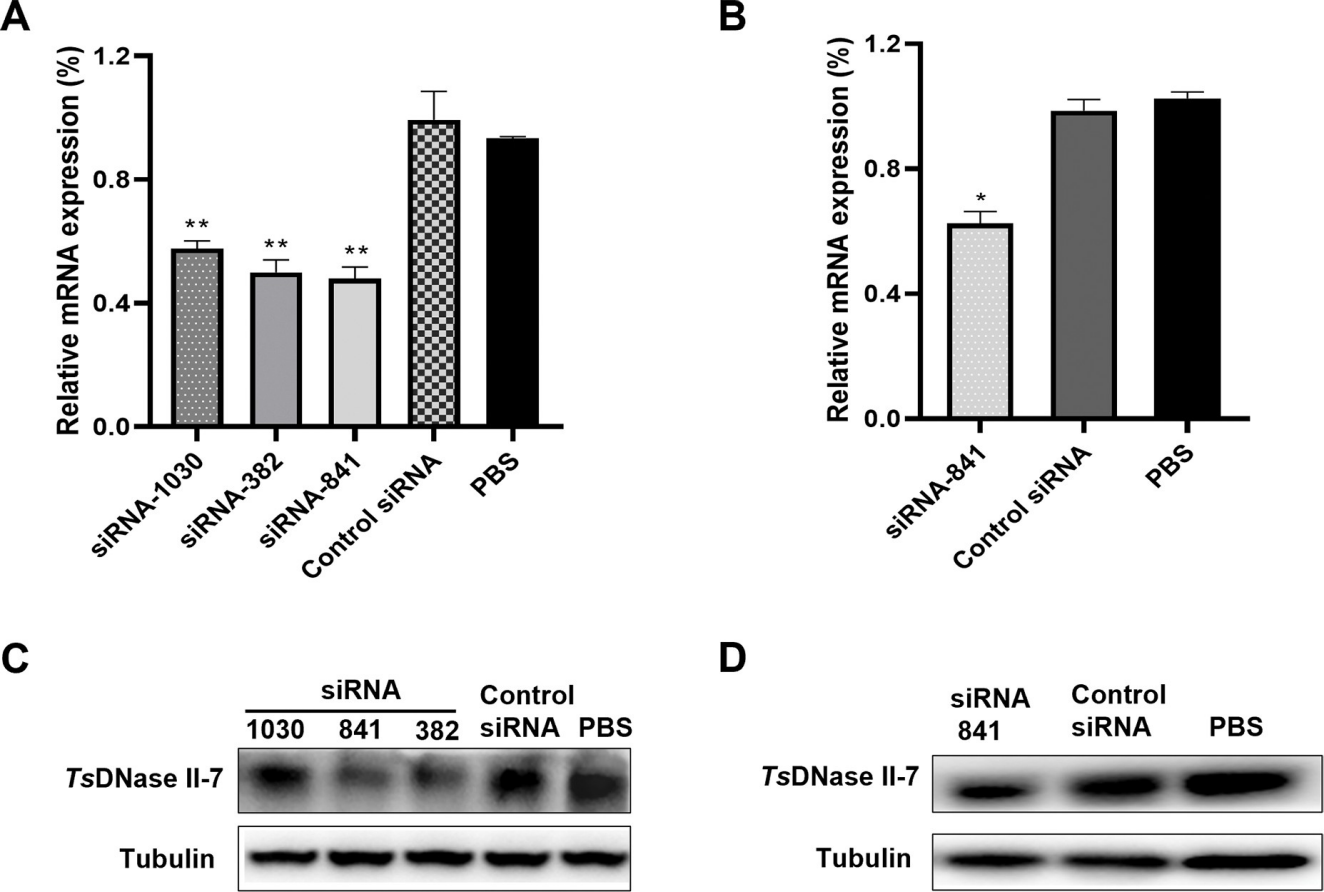
*Ts*DNase II-7 mRNA and protein expression levels in *T*. *spiralis* or siRNA-treated *T*. *spiralis*. (A) qPCR analysis of the relative transcription levels of the *Ts*DNase II-7 gene in ML with three siRNAs. (B) Ad3 was collected from mice infected with ML treated with *Ts*DNase II-7 siRNA-841, control siRNA or PBS. qPCR analysis of the relative transcription levels of the *Ts*DNase II-7 gene in Ad3 with siRNA-841. Total RNA was extracted from 3,500 MLs or 2,500 Ad3 worms. The amount of input total RNA for the first strand cDNA template synthesis (reverse transcription) reaction is 1μg. Equivalent to about 100 ng cDNA are needed to initiate qPCR. Assays were performed in triplicate, and the results are shown as the mean ± SD. * *P* < 0.05, ** *P* < 0.01 relative to the control siRNA and PBS groups. (C-D) Western blot with anti-*Ts*DNase II-7-specific antibodies showing the specific inhibition of *Ts*DNase II-7 protein expression in crude somatic extracts of Ad3 (D) or ML (C) treated with siRNAs.

To gain further insight into the functional significance of *Ts*DNase II-7 during *T*. *spiralis* infection, the expression of the target gene *Ts*DNase II-7 was knocked down via RNAi. The qPCR results showed that in comparison to the control group, the relative transcription level of the *Ts*DNase II-7 gene was reduced in ML treated with 2 μM siRNA-1030, siRNA-382, and siRNA-841 (*P* < 0.01) (Fig 5A). The results related to the efficacy of siRNA-treated ML knockdown showed that the knockdown efficiency achieved with 2 μM siRNA-841 was the optimal knockdown effect. Therefore, 2 μM siRNA-841 was used to optimize the working conditions. When Ad3 worms were collected from the intestine of mice with siRNA-841-transfected ML, the *Ts*DNase II-7 mRNA expression levels were inhibited in comparison to those of the PBS group (*P* < 0.05) (Fig 5B). The *Ts*DNase II-7 mRNA expression levels in the control siRNA group were not significantly changed compared with those in the PBS group.

The transfection of ML with siRNA-1030, siRNA-382 and siRNA-841 resulted in reductions in *Ts*DNase II-7 protein expression as compared to that in the control siRNA-treated ML or PBS group (Fig 5C). Compared with those in the PBS group, the expression levels of *Ts*DNase II-7 of Ad3 were reduced when Ad3 worms were collected from the intestine of mice with 2 μM siRNA-841-transfected ML (Fig 5D). No evident reduction in the expression of the housekeeping protein GAPDH was observed in the worms treated with any *Ts*DNase II-7 siRNA.

### *Ts*DNase II-7 contribute to the invasion of Ad3 into IECs

**Fig 6:**
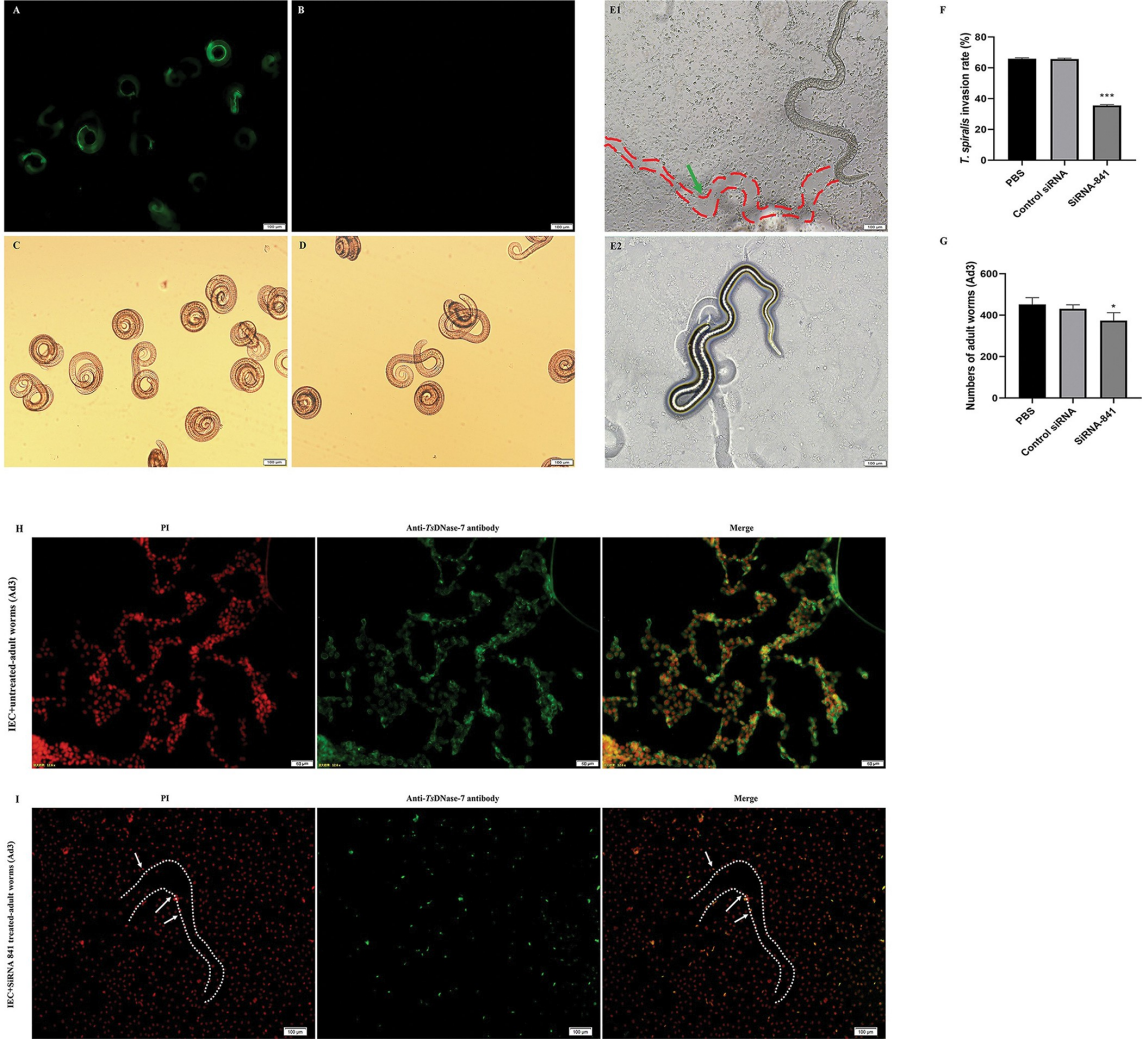
Invasion process of IECs by adult *T*. *spiralis* and RNAi inhibition of the Ad3 invasion of IECs. (A-D) Detection of fluorescent siRNA following the electroporation of *T*. *spiralis* ML. MLs were cultured for 24 h and exposed to FAM-labeled control siRNA by electroporation (A) or left untreated (B). Corresponding view of (Fig 6A and 6B) under a light microscope (C-D). The bar represents 100 μm in (A-D). E1: Ad3 that invaded the IEC monolayer was mobile, and its migrating trail was observed (shown as green arrow). The IECs invaded by adult *T*. *spiralis* were damaged. E2: The noninvaded Ad3 was still suspended in the medium and located on the surface of IECs. Scale bars, 100 μm in (E1 and E2). F: Inhibition of Ad3 invasion by siRNA-841 in IECs (Inhibition of siRNA-841 on Ad3 invasion of IECs). G: The number of adult worms recovered from mice infected with ML electroporated with siRNA-841 by electroporation. Data are presented as the mean ± standard deviation (SD) of three independent assays. * *P* < 0.05, *** *P* < 0.001 compared with the control siRNA-treated group and untreated group. (H-I) Microscopy of damaged cells and invasion of IECs by Ad3 treated with siRNA-841. After removing of agarose, the cell monolayer was stained with PI and observed under a fluorescence microscope. The nuclei of the damaged cells were intensely and uniformly stained red, showing the serpentine trail left by adult *T*. *spiralis* (shown as arrow). The antigen present on the damaged cells was detected with *Ts*DNase II-7 serum and visualized as green fluorescence. Compared with the untreated Ad3 group (H), little immunostaining of *Ts*DNase II-7 (green) was observed in the siRNA-treated group (I) because of the successful inhibition of *Ts*DNase II-7 expression. The scale bars are 50 μm in (H), and 100 μm in (I).

Twenty-four hours following electroporation by FAM-labeled control siRNA, fluorescent staining within the ML was observed by fluorescence microscopy, the untreated larvae did not display fluorescence staining (Fig 6A–6D), indicating that the siRNA was efficiently transfected into the worm body by electroporation.

Knockdown of *Ts*DNase II-7 mRNA by RNAi inhibited the Ad3 invasion of IECs *in vitro*. The *in vitro* invasion test was performed using Ad3 collected from mice inoculated with *T*. *spiralis* ML transfected with siRNA-841 to investigate the effect of *Ts*DNase II-7 on adult *T*. *spiralis* invasion. After incubation with the IEC monolayer in semisolid medium for 2 h, the invaded adult worms and noninvaded adult worms were observed and counted (Fig 6E and 6F). Additionally, knockdown of *Ts*DNase II-7 with siRNA-841 significantly suppressed Ad3 invasion of IECs, which exhibited a 35.54% decrease when the worms were collected from the intestine of mice with 2 μM siRNA-841-transfected ML (*P* < 0.001) (Fig 6F). A significant reduction was observed in the Ad3 invasion of IECs in mice inoculated with ML electroporated with siRNA-841 compared with that in mice inoculated with untreated ML or control siRNA (*P* < 0.05) (Fig 6G).

### *Ts*DNase II-7 protein is present around damaged cells invaded by adult *T*. *spiralis*

After the invasion assay, the *Ts*DNase II-7 protein was detected in damaged cells (stained with PI) using anti-r*Ts*DNase II-7 serum. Trails of damaged cells caused by Ad3 that migrated through the monolayers were left. These trails were documented by nuclear staining with PI (arrow, Fig 6I). Additionally, IFAs revealed that intense fluorescence staining was detected on the untreated-Ad3 invasion of IECs probed with anti-r*Ts*DNase II-7 serum (Fig 6H). However, little staining was observed on cells after they were incubated with Ad3 knocked-down with *Ts*DNase II-7-specific siRNA (Fig 6I). Proteins secreted from adult *T*. *spiralis* were also recognized in damaged cells (Fig 6), suggesting that *Ts*DNase II-7 was located in the adhesive trails and participated in the *T*. *spiralis* invasion of IECs.

## Discussion

Intestinal parasites, such as protozoa and nematodes, often infect mucosal surfaces of the intestinal tract, which are critical for their transit or residence within their host. IECs, which line the mucosal surfaces, seem to exist between the homeostatic balance maintained in the presence of commensal intestinal parasites and the necessarily destructive response to protozoa and nematodes that invade and cause infection in the immunocompromised host [21–22]. For example, the common intestinal protozoan parasites are *Cryptosporidium parvum* and *Giardia lamblia*. In fact, invasion is mediated by proteins such as cysteine proteases and galactose/N-galactosamine-specific lectin, leading to loss of intestinal barrier function and increased IEC death [23–24]. Additionally, during gastrointestinal nematode infection, ES products of adult *Haemonchus contortus* and *Teladorsagia circumcincta* disrupt the junctions of epithelial cells and increase epithelial permeability *in vitro* [25]. These changes also occur during *T*. *spiralis* infection [26].

The relevant literature indicates that several categories of enzymes from the adult *T*. *spiralis* worms, including cathepsin, nudix hydrolase, serine protease and serine protease inhibitor, have been identified during invasion of the host’s small intestinal epithelium [7, 27–29]. Among them, the function of cathepsin X in *T*. *spiralis* larval invasive capacity was identified using RNAi [29]. RNAi was first identified in the nematode worm *C*. *elegans* and has since been widely applied to the molecular characterization and functional analysis of parasites of medical-veterinary importance [30]. Examples of its application include the characterization of enolase A in *Taenia crassiceps* [31], and a matrix metalloprotease 12A in *Haemonchus contortus* [32]. In this study, we utilized RNAi, an excellent approach for expediting research in cell and molecular biology, to explore the function of *Ts*DNase II-7.

*T*. *spiralis* is associated with large amounts of secreted and soluble DNase II–like protein family members (an estimated 125), while none of which have classical nuclease active sites [14]. Therefore, the studies of *Ts*DNase II-7 have presented several unanswered questions regarding its biological function. The *Ts*DNase II-7 gene, a member of the DNase II family, is highly transcribed in intestinal AW stages [5, 33]. BLAST analysis revealed that adult specific *Ts*DNase II-7 belonged to the DNase II superfamily and demonstrated partial identity with sequences from a range of organisms, including parasitic nematodes, protozoa and mammals. The phylogenetic tree showed that *T*. *spiralis* DNase II has the closest evolutionary status to the *Trichinella* group (*T*. *pseudospiralis* and *T*. *nativa*), forming a separate and monophyletic clade. Our comparison of the *Ts*DNase II-7 sequence and enzyme activity analysis demonstrated that *Ts*DNase II-7 lacks traditional enzyme activity sites and nuclease activity. Furthermore, spatiotemporal specificity analysis confirmed that the *Ts*DNase II-7 gene was highly expressed in the adult stage (3 dpi-5 dpi). IFT with anti-r*Ts*DNase II-7 serum revealed that DNase II was homogenously distributed along the external surface of the *T*. *spiralis* Ad3 was mainly located at the cuticles and stichosomes. Accordingly, we speculate that the effect of *Ts*DNase II-7 from adult *T*. *spiralis* on IECs contributes to the invasion of the intestinal wall by the worm, but its specific role in the invasion process remains unexplored.

Generally, the methods to deliver siRNA into parasites to knock down parasite genes include immersion, electroporation, and microinjection. In this work, knockdown of the *Ts*DNase II-7 gene by the electroporation of specific siRNA-841 reduced the *Ts*DNase II-7 transcription and expression levels, indicating that the expression of both *Ts*DNase II-7 mRNA and protein could be knocked down by siRNA-mediated RNAi. Additionally, siRNA knock down of *Ts*DNase II-7 expression was stable in intestinal AW at 3 dpi after the mice were orally infected using siRNA-transfected ML. Current findings in the *in vitro* model suggest that the mechanism by which *T*. *spiralis* Ad3 invades IECs may involve both mechanical and biochemical damage. In fact, several studies have suggested that pathogens (e.g., *Plasmodium spp*.) usually invade multiple gut epithelial cells, and each of these cells undergoes cell death (apoptosis) following invasion [34]. Moreover, the estimation of cell death following invasion is an actual indicator for calculating the invasion efficiency [10, 35]. Previous studies have shown that attachment to host cells and invasion are mediated by proteins such as *Entamoeba histolytica*-cysteine proteases and Gal/GalNAc-lectins, galactose/N-galactosamine-specific lectin and *T*. *gondii*-SAG1 homologous proteins [24, 36–37]. Our results complement the above studies, showing that after downregulating *Ts*DNase II-7 in the adult stage of *T*. *spiralis*, the percent of invaded worms in IECs was substantially decreased. After worm invasion, we observed cells with apparent damage, which were slightly lessened when IECs were cocultured with adult worms treated with siRNA targeting *Ts*DNase II-7 by PI staining under microscopy. Knockdown of *Ts*DNase II-7 gene expression effectively suppressed *T*. *spiralis* invasion into IECs *in vitro*, confirming that *Ts*DNase II-7 participated in adult worm penetration of host intestinal tissue. To understand whether *Ts*DNase II-7 binds with various proteins of IECs, further experiments were necessary to verify which IEC proteins bind with *Ts*DNase II-7 using the pull-down assay, mass spectrometry and yeast two-hybrid system [38–39]. Previous studies had illustrated that oral vaccination with *T*. *spiralis* DNase II-1 vaccine delivered by attenuated *Salmonella* showed a significant immune protection against challenge in BALB/c mice [40]. *Ts*DNase II-7, as a homologue, may also have a better immune protective effect. These findings confirmed that *Ts*DNase II-7 plays an indispensable role in invasion and development and may be a new potential molecular vaccine target against *T*. *spiralis* infection.

Together, *Ts*DNase II-7 is highly transcribed and expressed in the adult stage of *T*. *spiralis*, and is mainly localized in the cuticle. *Ts*DNase II-7 promoted *T*. *spiralis* penetration into IECs, while knockdown of the *Ts*DNase II-7 gene by siRNA-*Ts*DNase II-7 impaired worm invasive ability. Our results highlight the power of investigating *T*. *spiralis* invasion in the context of a whole *in vitro* invasion model of IEC to explicate the mechanism of host-parasite interactions.
